# Uncovering *cis* Regulatory Codes Using Synthetic Promoter Shuffling

**DOI:** 10.1371/journal.pone.0002030

**Published:** 2008-04-30

**Authors:** Ali Kinkhabwala, Călin C. Guet

**Affiliations:** 1 Laboratory of Living Matter and Center for Studies in Physics and Biology, Rockefeller University, New York, New York, United States of America; 2 Systemic Cell Biology, Max Planck Institute for Molecular Physiology, Dortmund, Germany; 3 Institute for Biophysical Dynamics, University of Chicago, Chicago, Illinois, United States of America; Karolinska Institutet, Sweden

## Abstract

Revealing the spectrum of combinatorial regulation of transcription at individual promoters is essential for understanding the complex structure of biological networks. However, the computations represented by the integration of various molecular signals at complex promoters are difficult to decipher in the absence of simple *cis* regulatory codes. Here we synthetically shuffle the regulatory architecture — operator sequences binding activators and repressors — of a canonical bacterial promoter. The resulting library of complex promoters allows for rapid exploration of promoter encoded logic regulation. Among all possible logic functions, NOR and ANDN promoter encoded logics predominate. A simple transcriptional *cis* regulatory code determines both logics, establishing a straightforward map between promoter structure and logic phenotype. The regulatory code is determined solely by the type of transcriptional regulation combinations: two repressors generate a NOR: NOT (a OR b) whereas a repressor and an activator generate an ANDN: a AND NOT b. Three-input versions of both logics, having an additional repressor as an input, are also present in the library. The resulting complex promoters cover a wide dynamic range of transcriptional strengths. Synthetic promoter shuffling represents a fast and efficient method for exploring the spectrum of complex regulatory functions that can be encoded by complex promoters. From an engineering point of view, synthetic promoter shuffling enables the experimental testing of the functional properties of complex promoters that cannot necessarily be inferred *ab initio* from the known properties of the individual genetic components. Synthetic promoter shuffling may provide a useful experimental tool for studying naturally occurring promoter shuffling.

## Introduction

*Cis* transcriptional regulation is a powerful driving force in the evolution of function and form [1], [2]. The fact that organismal complexity does not scale with the number of genes in particular emphasizes the importance of *cis*-based control mechanisms as a source of the observed biological complexity. Promoters constitute the DNA-encoded nodes of complex transcriptional networks. However, within each promoter, transcriptional regulators (TR) themselves form *cis*-based networks of combinatorial interactions, similar to integrated computational devices [2]. Promoters are therefore DNA-based processing units that use TR inputs to integrate multiple metabolic and external signals into ON or OFF transcriptional outputs of specific genes. The biological information processing at the promoter level can be formally described with the computational language of logic functions [3], [4], which has been a powerful paradigm in understanding the regulation of developmental programs [5]. For example, the promoter of the classic *lac* operon, which is repressed by LacI and activated by CAP, can be described as an ANDN logic, expressing the *lac* genes if and only if lactose is the sole carbon source, with CAP bound and LacI not bound [4].

The quest for simple *cis* regulatory codes is therefore important but also challenging, given the difficulty in even identifying *cis* regulatory elements within the vast non-coding sequences of DNA [6]. In the ideal case, given knowledge of the binding sites for all transcription factors in a genome, one would like to predict what types of regulatory/computational functions can be performed at each individual promoter. For example, knowledge of the identity and position of a TR in *E. coli* is already a good predictor of the type of regulation (repression or activation) performed at a particular promoter [7]. On the other hand, such simple *cis* regulatory codes are very hard to uncover in the highly complex *cis* regulatory regions of eukaryotes [8]. A synthetic approach, which would be complementary to more classic genetics approaches, could prove helpful in revealing the complex *cis* regulatory codes available at single complex promoters.

Here we use such a synthetic approach [9] to study the combinatorial regulation of transcription at individual bacterial promoters, as first proposed in [10]. Specifically, we use the bacterial σ70 promoter of *E. coli* as a simple experimental model system to explore the ability of individual promoters to integrate multiple regulatory inputs, with the goal of uncovering simple rules or *cis* regulatory codes that may connect certain promoter architectures to their function.

## Results

### Design of synthetic promoter shuffling library

**Figure 1:**
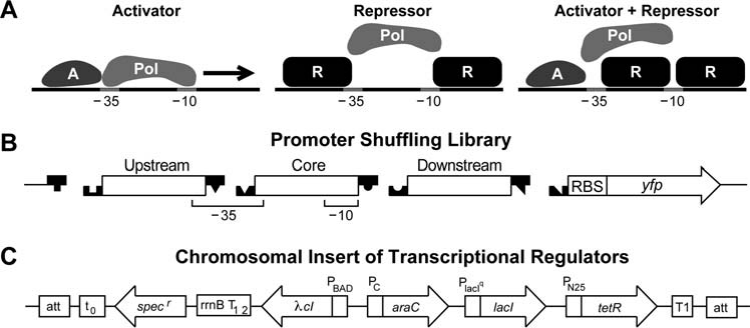
Description of synthetic promoter shuffling scheme. (A) Examples of promoters binding activator ‘A’ (left), repressor ‘R’ (middle) or both, activator and repressor (right). ‘Pol’ labels the RNAP. Activators nearly universally bind upstream of the −35 region in order to make direct, non-interfering contact with RNAP to help it bind. Repressors, on the other hand, can bind anywhere in the immediate region to successfully block RNAP binding. (B) Promoter shuffling scheme. We dissected the bacterial promoter into three regions encoding operator binding sequences (Upstream, Core, Downstream). The −35 and −10 RNAP binding sites and ribosomal binding site (‘RBS’) are indicated. The library consists of double-stranded DNA fragments with region-specific three-nucleotide overhangs allowing for ordered ligation to each other and, collectively, to a backbone vector. The complex promoter controls expression of a *yfp* gene. (C) Chromosomal insert of transcriptional regulators. *araC*, *lacI*, and *tetR* are transcribed from constitutive promoters, while *λcI* is regulated by an arabinose inducible promoter P_BAD_. Also indicated are the transcriptional terminators t_0_, *rrnB* T_1 2_, and T1; the gene for spectinomycin resistance *spec^r^*; and the λ phage attachment site, *attB*.

**Figure 2:**
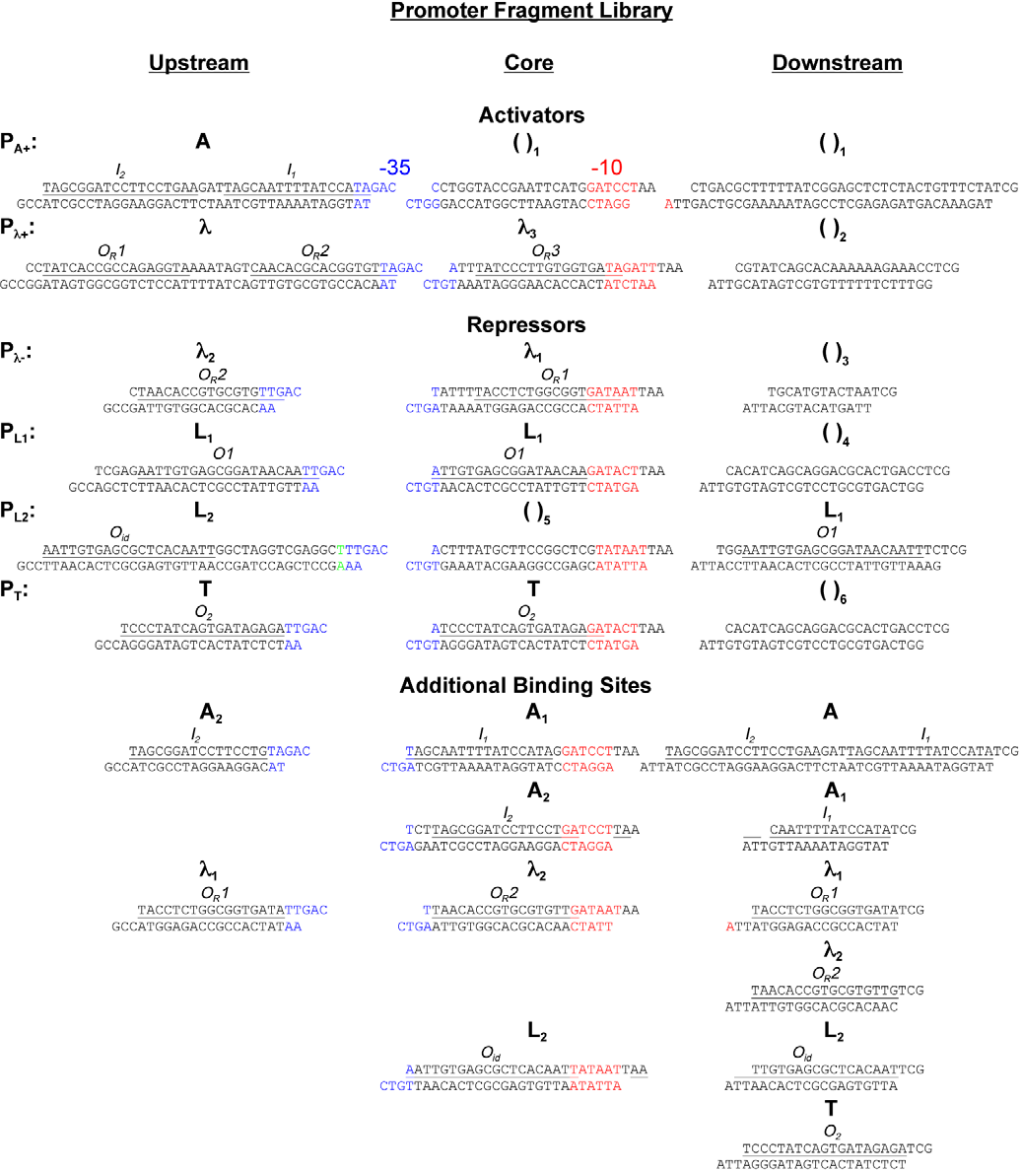
Promoter fragments used to construct combinatorial library. The first six rows correspond to the original promoters on which the library is based, the two activators: P_A+_ and P_λ+_, and the four repressors: P_λ-_, P_L1_, P_L2_, and P_T_. Promoter fragments ‘Upstream’, ‘Core’, and ‘Downstream’ of the −35 (blue) and −10 (red) regions are displayed. Each fragment has unique three-nucleotide overhangs, allowing properly-ordered assembly upon ligation to each other and the plasmid backbone. Binding regions of specific regulators are underscored and labeled. “Additional Binding Sites” refers to additional promoter fragments that were created to expand the library. The lone nucleotide in green upstream of the −35 site in P_L2_ indicates the accidental insertion of a ‘T’ when we designed this promoter fragment; it has negligible effect on the strength of repression by LacI.

Using a combinatorial synthesis approach [9], we shuffled multiple operator elements within the promoter region (Fig. 1A,B) to create complex regulatory architectures capable of executing diverse promoter-encoded logic (PEL) functions. For our library we used a wide range of operators for the well-characterized TRs AraC, LacI, λcI and TetR (Fig. 2). These regulators represent all known TR classes of *E. coli*: activators (AraC), repressors (LacI, TetR), and dual regulators (λcI) [7]. The genes for the four different regulators were integrated into the chromosome at the λ phage attachment site, *attB* (Fig. 1C). LacI and TetR were constitutively expressed, whereas λcI and AraC were under inducible control. Integration of these four regulators at a single locus in the bacterial chromosome allowed for greater consistency of expression than would be obtained with plasmid-based expression. This set of four TRs and their corresponding operators therefore form a simple and extremely well-characterized genetic system that is ideal for studying complex promoter structure-function properties.

For the canonical σ70 promoter of *E. coli*, the RNA polymerase binding sites define three modular promoter regions: upstream of −35; core, from −35 to −10; and downstream of −10 (Fig. 1A). For each region we designed short DNA oligomers with and without regulator binding sites that were flanked by region-specific overhangs (Figs. 1B and 2). We were then able to construct, through ligation, complex promoters that regulated the expression of a yellow fluorescent protein (YFP) gene (Fig. 1B). The logic phenotype of the complex promoters was determined by growing individual bacterial clones in the presence/absence of specific inducers (IPTG, aTc, and arabinose). The presence of the inducers changes the binding state of the TR at the promoters, enabling the switching between ‘On’ and ‘Off’ input states. The expression of the *yfp* gene serves as the output of the complex promoter, determining ON (transcription present) or OFF (transcription absent) output states of the individual promoters.

### Library of forward designed complex promoters

**Figure 3:**
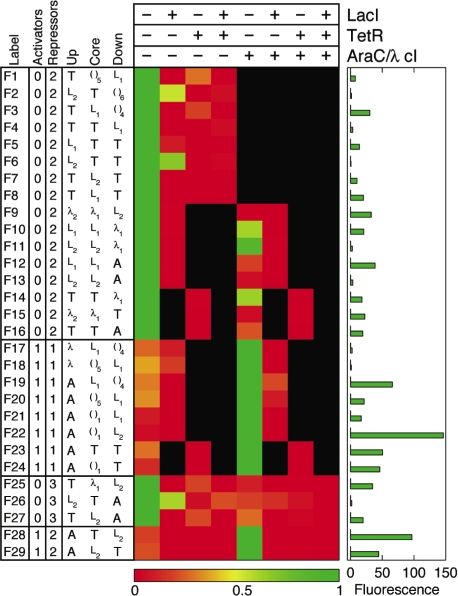
Promoter architectures and transcriptional logic phenotypes of the forward-designed promoter library. Rows represent different promoter architectures (F1–F29). The two columns labeled ‘Activators’ and ‘Repressors’ indicate the number of distinct activators and repressors capable of binding each promoter. Columns labeled ‘Up’, ‘Core’, and ‘Down’ indicate the three specific DNA fragments coding for various operators (see Fig. 2 for fragment sequences). Parentheses indicate a sequence lacking TR binding sites. For each promoter architecture, gene expression levels are represented by fluorescence measured for individual clones grown in eight wells corresponding to all eight different conditions of binding/nonbinding (+/−) of LacI (L), TetR (T), and AraC/λcI (A/λ). For clarity, we show only the relevant growth conditions for each promoter (expression levels were dependent only on the presence/absence of regulator specific inducers). Fluorescence was determined at an optical density (600 nm) of 0.3. Each row is normalized to the minimum (‘0’, red) and maximum (‘1’, green) fluorescence values for that particular promoter, with the actual minimum and maximum values given in the accompanying histogram to the right (all minimum values were very low and consistent with control cells lacking the *yfp* gene).

**Figure 4:**
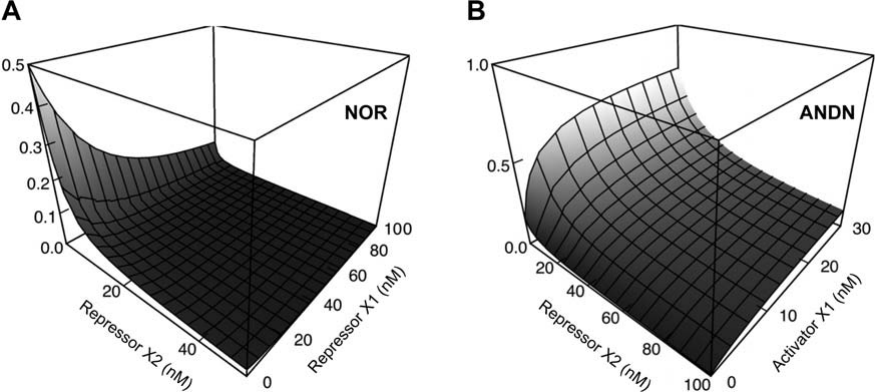
Simple thermodynamic models of NOR and ANDN logic phenotypes. (A) Two repressors generate a NOR logic. (B) One repressor and one activator generate an ANDN logic. The model assumes non-interactive regulators that compete with (repressors) or help (activators) RNA polymerase bind to the promoters (see Materials and Methods for details). As the concentration of the active repressors or activators (x and y axis) is varied, the transcription of the gene they control changes (z axis).

We designed in a combinatorial fashion 29 complex promoters that utilize diverse architectures that were expected to sample well the total space of logical phenotypes (Fig. 3). Our expectations were based on the simple assumption that regulator binding at a repressor binding site, no matter its position, would always generate repression, whereas regulator binding to an activator binding site would lead to activation only if positioned upstream of the −35 site and would otherwise lead to repression. A simple thermodynamic model based on these assumptions shows that in the absence of cooperative interactions, a complex promoter with binding sites for two different repressors should implement a NOR logic, whereas a complex promoter with binding sites for an activator and a repressor should implement an ANDN logic (Fig. 4). Fluorescence values for bacterial clones containing the 29 complex promoters were measured for the eight different inducer conditions corresponding to the binding/non-binding (+/−) of LacI, TetR, and AraC/λcI (Fig. 3). The physical presence or absence of regulators at operators represents a natural way to define the ‘On’ and ‘Off’ input states [11].

By shuffling the three regions of the bacterial promoter with our operator library, we constructed several promoters implementing the Boolean operations NOR and ANDN for two inputs (binding sites for two distinct transcriptional regulators, F1–F24) and three inputs (binding sites for three distinct transcriptional regulators, F25–F29) (Fig. 3).

### A simple *cis* regulatory code determines logic phenotype

For *E. coli* promoters binding only one regulator, it is well known that the identity of the TR and position of its operator determines whether the TR will activate or repress the transcription of a gene [7]. Our results demonstrate that such simple yet powerful principles can be extended to more complex promoters as well: a combination of two repressors results in a NOR type PEL (F1–F16), whereas a combination of a repressor and an activator produces an ANDN type PEL (F17–F24). This general principle extends as well to three input promoters: three repressors confer a three-input extension of the NOR phenotype (F25–F27): NOT (a OR b OR c), whereas two repressors and one activator results in a three-input extension of the ANDN phenotype (F28–F29): a AND NOT (b OR c).

Each of the two PEL functions, NOR and ANDN, are implemented in a multitude of operator combinations at the individual complex promoters (Fig. 3). Therefore the logic phenotype does not depend on any specific combination of particular regulators, but only on the type of regulation the transcriptional regulators perform, either positive or negative. As long as two different repressors are present, the complex promoters function as NOR logics, and alternatively, when a repressor and an activator are both present, the complex promoters encode an ANDN logic.

Differing binding site strengths do not affect the logic type, as can be seen in Fig. 3 by comparing promoters constructed from ‘L_1_’ (weaker) versus ‘L_2_’ (stronger) binding sites (e.g., F7/F8, F12/F13, F21/F22). The number of binding sites for a particular regulator in general does not affect the logic type (e.g., TetR binding in F1/F4, F23/F24). However, in a few cases the presence of only one operator site for a particular repressor results in leaky repression (e.g., λ_1_ in F10, F11, F14 and L_2_ in F6 and F26). It is remarkable that TR binding at any of the three promoter regions effectively generates repression (e.g., LacI binding of L_1_ in F1, F3 and F5). This more generally demonstrates a degree of robustness of PEL functions to certain types of promoter shuffling.

### Dynamic range of regulation of complex promoters and fuzzy logic behavior

**Figure 5:**
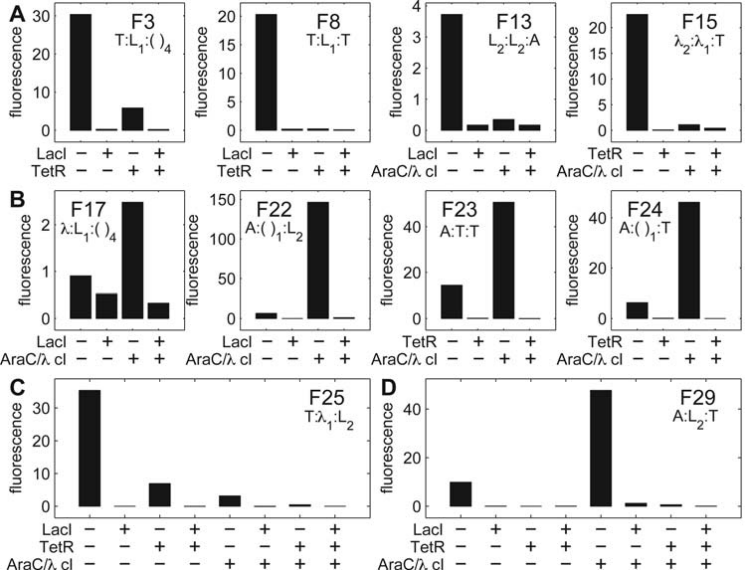
Bar plots of specific promoters shown in Fig. 3. (A) Two-input NOR promoters. (B) Two-input ANDN promoters. (C) Three-input NOR promoter. (D) Three-input ANDN promoter.

Our complex promoter library displays a wide range of promoter strengths and leakiness of each logic type as can more clearly be seen in the fluorescence histograms of selected promoters (Fig. 5). The ratio of the ON state to the leakiest of the OFF states provides a convenient quantification of the leakiness or fuzziness of individual promoters. For example, F3 is a stronger promoter than F8, but is a more fuzzy NOR logic (F3 has an ON/OFF ratio of 5.2 while the ratio for F8 is 75). While the range of the ON state to the leakiest OFF state is up to 150 fold (F7), the ratio of the ON state to the tightest OFF states ranges over four orders of magnitude (F22) or higher, since many OFF states are indistinguishable from the autofluorescence of the control cells lacking the *yfp* reporter gene. This wide regulatory range is remarkable, given the fact that our library contains only five different −10 and −35 RNA polymerase binding sites (Fig. 2). The combinatorial potential of this collection of PELs therefore demonstrates the rapid ability of the combinatorial approach to find an optimal promoter architecture for a given logic phenotype with respect to strength, leakiness, and dynamic range of the ON/OFF transcriptional states. These aspects are important since a gene regulated by a complex promoter could trigger downstream activities differentially as a function of its absolute concentration, similar to a fuzzy or multi-valued logic device [12]. For example, F23 could work either as a two-input ANDN logic or as a simple one-input ON/OFF gate with respect to TetR binding. Overall, it is noteworthy that these 29 promoters, which represent only a small subset of the few hundred possible combinations of operators in our library, still sample such a wide span of promoter strengths and wide dynamic range of transcriptional regulation.

In Fig. 3, a handful of the complex promoters display poor logic phenotypes with the ratio of the lowest ON state to the highest OFF state less than two (F2, F6, F10, F11, F14 and F26). The reason for this is the presence of one of two particularly inefficient operator elements: L2 at the upstream position (F2, F6, F26) and the presence of a single λ operator at the downstream position (F10, F11, F14). These two elements do not provide enough repression at the indicated positions in the absence of another binding site for the respective TR at another site in the promoter. In the case of L2, the fact that the operator site is positioned some distance away from the −35 RNAP binding site (15 nt away) explains the poor repression of LacI when bound solely at this position of the promoter, since it cannot effectively physically hinder RNA polymerase from binding. The inability of λcI to effectively act as a repressor when bound to a single operator at the downstream position is most probably caused by its inability to effectively compete with RNA polymerase binding at this position [13].

### Library of randomly assembled complex promoters

**Figure 6:**
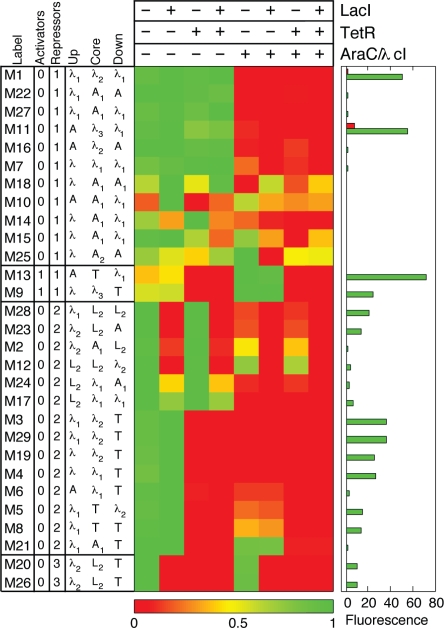
Randomly assembled promoter library. Color chart of 29 promoters created through random assembly (same plotting conventions as in Fig. 3). There are three sets of identical sequences (M14/M15, M3/M29, and M20/M26), leaving 26 unique sequences all different from the forward-designed sequences shown in Fig. 3. Promoters are grouped according to effective behavior of components, ordered from the cleanest implementation of the logic to the fuzziest.

In addition to the promoters designed in Fig. 3, we also constructed a library of randomly assembled complex promoters, yielding an additional 26 unique promoters, out of which only a fraction had more than one unique TR input (Fig. 6). Two additional promoters with effective ANDN logical phenotypes were found (M13 and M9), as well as thirteen additional promoters with effective NOR phenotypes (M28, M23, M2, M12, M24, M17, M3/M29, M19, M4, M6, M5, M8, M21). A three-input NOR was also present (M20/M26).

This set of randomly assembled NOR and ANDN promoters displayed similar properties to the forward designed library promoters. Most promoters in the randomly assembled library follow the same simple *cis* regulatory codes: two repressors determine a NOR logic, whereas one repressor plus one activator determines an ANDN logic. For those that fail to obey this code, the leakiness of the states that fail to conform to the expected logic type can be traced again to two operator elements, single λ operators downstream and upstream (M12, M20, M21) and single L_2_ operators upstream (M17). It is noteworthy that single λ operators can function as effective repressible elements, as can be clearly seen for M28 where the upstream λ_1_ operator effectively represses the promoter. The −10 site for RNA polymerase in M28 is very different from the −10 RNAP site in M8, while the −35 RNAP sites are identical. This difference in −10 sites may explain why in M28 the λ repressor can outcompete RNAP binding. In addition, the presence of a single binding site for AraC in M21 is not enough to compete with RNAP at the core position. M21 is the only example from both libraries of an operator positioned at the core position that does not effectively interfere with RNAP binding. This is indicative of the fact that single AraC molecules cannot effectively act as repressors unless they form loops or an additional nearby operator site is present for a second AraC molecule to cooperatively bind [14].

For both libraries of complex promoters (Figs. 3 and 6), logical phenotypes generally follow simple *cis* regulatory codes. The two *cis* codes are determined solely by activation and repression due to different regulators based on the presence (and position) of particular regulator binding sites in the promoter structure. This straightforward outcome is due to the simpler transcriptional regulation in prokaryotes. By contrast, transcriptional regulation in eukaryotes is expected to be significantly more complex due to long-range effects arising from chromatin-embedded *cis* regions as well as the many factors that constantly reshape chromatin structure and lead to epigenetic transcriptional states.

## Discussion

In the present study we have extended the use of combinatorial synthesis, originally employed to construct genetic networks composed of several cross-regulating transcriptional regulators [9], to the construction and analysis of individual complex promoters (as proposed in [10]). In general, the combinatorial synthesis of biological networks using simple and well-characterized genetic elements is a powerful tool for producing and sampling the phenotypes of large numbers of biological networks.

As noted in the introduction, complex promoters form intricate networks of interactions among TRs and RNA polymerase. These interactions result in complex computations at the level of the promoter. The breadth and complexity of computations that can be performed at this level of biological organization, the promoter level, should also impact the organization of other genetic and biochemical networks in the cell [15].

Surprisingly, the phenotypes of the complex promoters we obtained can be understood in terms of basic rules of repression and activation of transcription by individual TRs. This result is in contrast to the genetic networks obtained through combinatorial synthesis, where logic phenotypes for networks emerged that could not be reduced to the sum of the known interactions among the ingredient genetic components composed of genes for TRs and their promoters [9].

Because complex promoters in our library follow elementary repression and activation, we were able to uncover a simple *cis* regulatory code: two repressors code for NOR, and one repressor and an activator code for ANDN. Intriguingly, the NOR gate (along with NAND) is classified as a “universal gate” in computer science, with combinations of NOR gates capable of coding for any Boolean-type logic. The simple transcriptional code seems to also apply to three different TRs: three repressors generically lead to a three-input extension of the NOR, and two repressors plus an activator generates a three-input extension of the ANDN. The code breaks down in only a few cases in which the individual promoters contain just a single weakly binding operator for a particular TR that was positioned either upstream or downstream of the core region, where a single TR cannot effectively compete with RNAP binding. In all instances where two operator binding sites for a given TR are present, the repressors always manage to effectively bind and outcompete RNAP binding.

**Figure 7:**
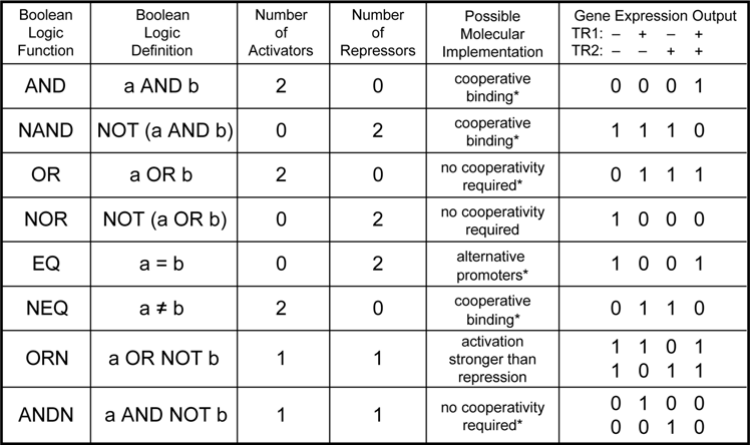
Two-input Boolean logic functions at the single promoter level in bacteria. Boolean logic functions are listed in the first column with their corresponding formal definitions given in the second column. The next two columns indicate the number of distinct activators and repressors required to generate the logic phenotype. The second to last column displays various molecular schemes mostly proposed by Buchler *et al.* [11] (marked by an asterisk) for implementing specific Boolean logic function at complex promoters. Many of these logic functions require intricate molecular schemes involving either regulator cooperativity or the presence of alternative promoters [11]. Hermsen et al [16] have recently proposed molecular implementations based on cooperative/competitive binding of several TRs for all of these logics. The last column displays Boolean output as a function of the binding states of two transcriptional regulator, TR1 and TR2, inputs at the promoter: ‘+’ (bound) and ‘−’ (not bound). Collectively, these functions represent a complete set of all two-input Boolean functions having outputs that depend on the state of both inputs.

The complex promoters in this study are characterized by simple interactions among the TRs: there are no cooperative interactions among different species of TRs, and there is no overlap between their binding sites at the operator level. Therefore, no PEL phenotypes were expected to arise from the cooperative or competitive binding of different TRs. Nontrivial cooperative and competitive binding are prerequisite mechanisms for encoding certain types of PEL [11], [16] as can be seen in Fig. 7. However, our method can be easily extended to the study of such complex interactions within complex promoters, which should allow for other types of logic behavior, such as NAND, EQ and NEQ [11]. Synthetic promoter shuffling can also be used to test various models for logic computation based on overlapping TR binding sites at complex promoters [16] in an experimentally comprehensive fashion.

Besides the study of prokaryotic promoters, synthetic promoter shuffling could also in principle be used to study the complexity of eukaryotic promoters. While the organizational complexity introduced by chromatin seems daunting, it might still be possible to learn how to incorporate chromatin effects into the design of synthetic promoter shuffling schemes in simple eukaryotes such as yeasts. By using unique overhangs, one can easily extend the number of shuffled genetic elements that can be ligated in a controlled fashion to obtain promoters with a larger number of transcription factor binding sites that can be shuffled in a combinatorial fashion. Starting from libraries based on simple non-cooperative interactions among TRs one could gradually include more complicated interactions to uncover other possible *cis* regulatory codes.

In recent years, *cis* regulation has been increasingly recognized as an important means by which biological systems evolve [1], [17]. Synthetic combinatorial promoter libraries could serve as useful experimental frameworks for studying the evolution of *cis* control. Though new regulator binding sites have long been assumed to primarily evolve by gradual point-wise mutations [18], regulator binding site rearrangements, or “promoter shuffling” (in analogy to the more familiar “exon shuffling”), could allow for more rapid and efficient exploration of the regulatory space of promoters and might therefore be an important evolutionary force [19]. Given the sequence similarity of many promoter elements, homologous recombination mechanisms could be expected to strongly contribute to promoter shuffling, in addition to insertion or deletion events promoted by mobile genetic elements. Promoter shuffling has recently been observed in higher eukaryotes [21], [22], and implicitly through the genomic reorganization of some bacteria [23], the presence of transposable elements in *Drosophila* heat shock promoters [24], and the structure of *cis* regulatory elements in vertebrates [25]. As demonstrated by synthetic promoter shuffling in our simple experimental system, shuffling of regulator binding sites can indeed lead to dramatic changes from one logic type to another (e.g. F16 to F23 and F26 to F28). On the other hand, many regulatory architectures in our library have logical phenotypes that are robust to shuffling (e.g. F4 to F5 and F28 to F29), demonstrating a balance between phenotype evolvability and robustness. The PELs in our library do not allow for cooperative hetero-protein interactions. Such non-interacting architectures may represent essential stepping stones in the course of promoter function evolution, with fine-tuned protein-protein interactions arising only at later steps. The traditional study of natural transcriptional networks primarily provides insight into the later, more refined stages of evolution, whereas synthetic promoter shuffling may provide greater insight into the early stages of promoter evolution, where the predominance of only a few control functions, such as NOR and ANDN, could be very important.

The power of synthetic promoter shuffling lies in the use of well-characterized genetic elements to explore the presence of possible *cis* regulatory codes and to assess the overall computational capacity of *cis* regulatory regions.

## Materials and Methods

### Chromosomal insertion of a cassette of transcriptional regulatory genes

Chromosomal insertion of the genes for the regulator proteins (Fig. 1C) was carried out as follows. We PCR amplified λcI from pZS21-λcI with flanking SphI (5′) and HindIII (3′) and inserted it into the SphI/HindIII sites of pBAD33:gfp(LAA) [26], thus replacing gfp(LAA). We then PCR amplified from this plasmid the fragment containing AraC-P_C_-P_BAD_-λcI (P_C_-AraC is inverted) using primers containing a flanking BstXI site (encoding a SphI-compatible overhang) and a flanking AatII site. After restriction with AatII and BstXI, we ligated this fragment to the AatII/SphI-restricted pZS4-lacI-tetR-Int [27] to make pZS4-λcI-araC-lacI-tetR-Int. We tested that the regulators functioned as expected by co-transforming the plasmid along with a set of plasmids containing promoters regulated by the four regulators (AraC, LacI, λcI, and TetR) controlling YFP.

We used the chromosomal integration method of [28] to integrate pZS4-λcI-araC-lacI-tetR-Int into the *attB* site of DH10B. Colonies with integrants were selected on spectinomycin plates and the integration of the transcriptional regulators cassette was confirmed by PCR. We designate this strain as λALT. We chose DH10B as the starting strain because it has a functional arabinose transport system (*araE*+ and *araFGH*+), yet does not metabolize arabinose (*araB*−, *araA*−, *araD*−).

### Oligonucleotide fragments used for complex promoter library

All single-stranded oligonucleotides that we used to construct our complex promoters are shown in Fig. 2. They were synthesized by Integrated DNA Technologies (Coralville, IA) with phosphorylated 5′ ends and ranged in size from roughly 30 to 45 bases. The upper and lower strands were mixed at equimolar concentrations, heated to 95° C, and then annealed by gradual cooling to room temperature to form double-stranded promoter fragments, with each fragment having specific 3-base overhangs that corresponded to its particular insertion region (Upstream, Core, Downstream) and allowed for ordered ligation.

Sequences for P_λ+_, P_λ−_, P_L1_, P_L2_, and P_T_ are based on those used in [9]. For the P_A+_ promoter, we employed the two activator AraC-binding sites (I_2_ and I_1_) leftward of the −35 [29], [30].

Our choices for the positions of the specific three-nucleotide overhangs regions used for ligation, which must be the same for all region-specific fragments, were governed by the following considerations. In order to allow for arbitrary activator sequences, for which transcriptional efficiencies can be highly susceptible to positional shifting and mutation, we decided to leave the region upstream of −35 unspecified. Similarly, due to the short segment between the −35 and −10 regions (typically 17 nucleotides long), a fixed three-nucleotide site would further limit the size of unique binding regions in that area. For these reasons, the break point was introduced inside the −35. We chose the central GAC in the −35 to be the same for all promoters, because it was the most conserved of all the possibilities. This required changing the wild-type TAC sequence to GAC in both the A+ and λ+ activator sequences, which, unfortunately, also increased their OFF-state leakiness. The other break point in the area around the −10 region was less restrictive because the region downstream of −10 can accommodate TR binding sites at various positions. For this reason, we simply specified that all promoters have the sequence TAA immediately downstream of their −10 sequence.

### Backbone plasmid

The backbone plasmid for the complex promoter library was constructed as follows: The *bla* gene together with its promoter was PCR amplified from pZS*1R-gfp. The leftward primer contained an XhoI site in addition to the DraIII site CACCGGTGG. The rightward primer contained an EcoRI site and the DraIII site CACTCGGTG. The underlined nucleotides represent the three nucleotide overhangs that result upon restriction with DraIII. The *bla* gene was cloned into the XhoI/EcoRI sites of pZA21-yfp (p15A origin of replication) replacing the promoter of this plasmid with the *bla* gene. The resulting vector pZA2-DraIII-bla-DraIII-yfp was cut with DraIII, resulting in a fragment with unique three nucleotide overhangs into which the complex promoter library was cloned. Sequencing later revealed that the EcoRI site of pZA2-DraIII-bla-DraIII-yfp was corrupted (replaced by the sequence GCTTAAGGCC). This had no noticeable affect on the downstream ribosomal binding site or the expression level of YFP.

### Complex promoter library assembly

For the forward-designed library, equimolar concentrations of specific, annealed, double-stranded oligonucleotide promoter fragments (one per region) were ligated to each other in the presence of the backbone (DraIII-cut plasmid pZA2-DraIII-bla-DraIII-yfp). For the randomly-mixed library, equimolar concentrations of multiple oligonucleotide fragments for each region were ligated in the presence of the backbone vector (see the final section below). In both cases, cells were electroporated into λALT strain and selected on LB+Kan plates.

Triplicate sequencing of five of the complex promoters, collectively sampling 12 unique oligonucleotide fragments, revealed no mutations, suggesting a high level of fidelity for synthesis of the oligonucleotides (as well as their annealing and ligation). Similar high-fidelity sequencing results for the random library are described below in the final section on the randomly mixed library.

### Growth medium

We used the following defined minimal medium due to its low background for YFP fluorescence measurements: 0.5 g (NH_4_)_2_SO_4_, 5.25 g K_2_HPO_4_, 0.225 g MgSO_4_·7H_2_O, 19 mg EDTA, 2.5 mg FeSO_4_ in 500 mL H_2_O adjusted to pH 6.8 with 85% H_3_PO_4_. The medium was filter sterilized and supplemented with 0.5% glycerol and 0.5% casamino acids.

### Fluorescence measurements

Individual colonies were grown overnight in the above defined medium along with spectinomycin (25 µg/mL) and kanamycin (30 µg/mL) in 96-well U-Bottom polystyrene plates (Becton Dickinson Labware, Franklin Lakes, NJ; BD Falcon 351177) on a microtiter plate shaker at 30° C. Overnight cultures were diluted by a factor of 1370 into fresh medium in eight different wells, representing all eight combinations of the three inducers arabinose, aTc and IPTG. The following concentrations of inducers were used: 0.1% arabinose, 100 ng/ml aTc, and 1 mM IPTG.

Well-sampled optical density (OD) and fluorescence growth curves were taken using a Victor Wallac2 multi-well fluorimeter (Turku, Finland). OD was measured at 600 nm (10 nm bandpass, integration time 0.1 s). YFP fluorescence was measured using the following instrument settings: CW-lamp excitation filter, HQ505/10x (centered at 505 nm with a 10 nm bandpass); emission filter, F535 (centered at 535 nm with a 25 nm bandpass); CW-lamp energy, 7000; integration time, 0.3 s; emission aperture, damp; counter position, top. Fluorescence vs. OD curves were plotted and interpolated using a Hermite polynomial method in Matlab called “fchip” (The Mathworks, Inc., Natick, MA). All fluorescence values used in this paper were measured around OD = 0.3, however the qualitative logic phenotype did not change throughout the growth curve. Fluorescence values were background-subtracted (to account for autofluorescence of the cells), and divided by 1000.

The background was estimated as follows. λALT cells lacking the complex promoter plasmid were used as a control. They were grown exactly as complex promoter cells except for the absence of kanamycin in the medium. Background fluorescence signal for the control cells was observed to decrease as a function of OD due to cell turbidity. Background fluorescence was similarly interpolated at an OD of 0.3, as explained above, for each well position, and has been subtracted throughout this paper. The lowest level of fluorescence for each forward-designed and randomly-mixed promoter (excluding the leaky promoters M1 and M11, see Fig. 6) was consistent with the control cell background fluorescence (1σ = 0.11), implying very tight control (low leakiness). A quadruplicate growth assay of a subset of the library showed that well-to-well variations were less than 5%, consistent with expected levels of pipetting errors. This was similar to well-to-well variations of background fluorescence of the control cells (that lacked the plasmid containing *yfp*).

### Randomly mixed library

We have combined all the fragments shown in Fig. 2, excluding only those few that do not have a repressor or activator binding site. The only bias applied to the library was a favoring of the presence of either of the two activators (P_A+_ or P_λ+_) in the ‘Upstream’ region by using equimolar concentrations for these two pieces that together equaled the sum of all concentrations of the other elements in the ‘Upstream’ region. Everything else was added in strictly equimolar proportions. However, each double-stranded oligonucleotide sequence has its own ligation efficiency that will of course also contribute to the bias of the library.

Despite these unknowns, we obtained a fairly heterogeneous library. Twenty-nine randomly-picked clones are displayed in Fig. 6, along with their corresponding promoter architecture revealed upon sequencing. There is no overlap with the forward-designed library described above, only three sets of clones, M14/M15, M3/M29, and M20/M26, turned out to be identical. Detailed analysis of the sequences revealed evidence for only a single mutation (an insertion in the middle of the λcI binding site in M21), further confirming the overall high fidelity of fragment synthesis and promoter construction.

### Simple thermodynamic model of NOR and ANDN logic

We first consider the case of two non-interacting repressors. The steady state behavior is determined by the thermodynamic binding constants: *K_R_*, *K*_1_, and *K*_2_ respectively specifying the independent binding strengths of RNA polymerase, regulator 1, and regulator 2 to the promoter. This gives:
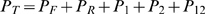
(1)

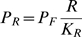
(2)

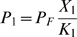
(3)

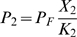
(4)

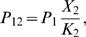
(5)where *R* denotes the RNA polymerase concentration; *X*_1_ and *X*_2_ denote the concentrations of regulators X1 and X2; and *P_T_* gives the total promoter concentration (which is present on a multi-copy plasmid) comprised of *P_F_*, *P_R_*, *P*_1_, *P*_2_, and *P*_12_ respectively denoting unbound, RNA polymerase bound, regulator X1 bound, regulator X2 bound, and regulators X1 and X2 bound promoter concentrations. The steady-state probability of having RNA polymerase bound at a single promoter (the “active” state) is:
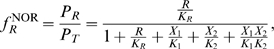
(6)which, in dimensionless units, is just:
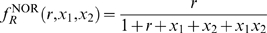
(7)with *r* = *R*/*K*, *x*_1_ = *X*_1_/*K*_1_, and *x*_2_ = *X*_2_/*K*_2_.

Taking regulator X1 to be an activator leads to a similar set of equations but with Eq. 1 changed to:
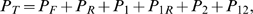
(8)to account for the additional species:
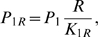
(9)which tracks the concentration of promoters with bound activator that have recruited RNA polymerase. *K*_1*R*_ is the binding constant describing the interaction of RNA polymerase with an activator-bound promoter. (For simplicity, we neglect other possibilities for the formation of promoters bound with both activator and RNA polymerase, including promoter-bound RNA polymerase recruitment of activator and the binding of pre-formed activator-RNA polymerase complexes to the promoter.) Here, the probability of RNA polymerase being bound at a given promoter (the “active” state) is:
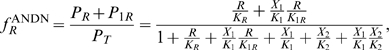
(10)which can be expressed dimensionlessly as:
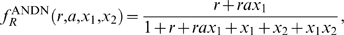
(11)where *a* = *K_R_*/*K*_1*R*_ denotes the additional affinity of RNA polymerase for binding of the promoter due to the bound activator.

In Fig. 4, we plot these probabilities in the more intuitive actual concentration units to highlight the general skewed nature of the distributions arising from differing interaction strengths and differing physiological concentration ranges. The values we used for the displayed NOR gate were as follows:
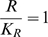
(12)

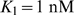
(13)

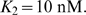
(14)Similarly, the values used for the displayed ANDN gate were as follows:
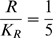
(15)

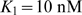
(16)

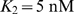
(17)

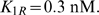
(18)

